# Current Knowledge of the Impact of Vitamin D in Coronary Artery Disease

**DOI:** 10.3390/ijms26115002

**Published:** 2025-05-22

**Authors:** Freja Esager Jespersen, Daniela Grimm, Marcus Krüger, Markus Wehland

**Affiliations:** 1 Department of Biomedicine, Aarhus University, 8000 Aarhus, Denmark; 202109344@post.au.dk (F.E.J.); dgg@biomed.au.dk (D.G.); 2 Department of Microgravity and Translational Regenerative Medicine, Otto von Guericke University, 39106 Magdeburg, Germany; marcus.krueger@med.ovgu.de

**Keywords:** vitamin D, cardiovascular disease, coronary artery disease, cholecalciferol, randomized controlled trials

## Abstract

Coronary artery disease and vitamin D deficiency are both widespread conditions with a high incidence worldwide. Coronary artery disease is a complex illness with variable manifestation and pathogenesis. It often involves the development of atherosclerosis, and it frequently has serious or even fatal consequences for the patient. Vitamin D receptor expression is found in many tissues throughout the body, which results in a broad effect of the vitamin. Studies have found correlations between vitamin D deficiency and the development of coronary artery disease as well as other cardiovascular diseases, such as hypertension. This review will discuss randomized controlled trials conducted from 2020 forward, aiming to elucidate whether vitamin D supplements have the potential to be used as an add-on treatment for coronary artery disease. The randomized controlled trials all used vitamin D as intervention and tested a population suffering from coronary artery disease or the risk of developing it. Even though animal studies found evidence that vitamin D can regulate inflammation, lipid profile, foam cell formation, vessel reactivity, and blood pressure, which are all mediators in the development of atherosclerosis, the results from the randomized controlled trials were ambiguous. The general older population did not seem to benefit from the treatment, but different subgroups such as patients with type 2 diabetes and patients with more developed coronary artery disease exhibited some positive effects from the treatment. Furthermore, vitamin D showed cardioprotective effects following coronary artery bypass surgery, which make it a possible add-on treatment before invasive coronary intervention. The question in focus still needs further research and a more focused approach on subgroups that may benefit from treatment.

## 1. Introduction

### 1.1. Coronary Artery Disease (CAD)

Cardiovascular diseases are affecting numerous people around the world. In 2022, the prevalence rate of ischemic heart disease globally was 3610.2 per 100,000 and the death rate was 108.8 per 100,000. Furthermore, ischemic heart disease had the highest rate of age-standardized disability-adjusted life years (DALYS) globally [1].

Coronary artery disease (CAD) results mainly from the formation of atherosclerotic plaques in the coronary arteries. The progression of atherosclerosis narrows the available artery lumen, which leads to a reduction in blood supply to the myocardium and thereby ischemia of the heart [2]. Ischemic heart disease can, however, also be the result of disease in the coronary arteries without obstruction [3]. In its chronic phase, CAD can manifest in patients as stress-induced angina, other chest discomfort or apnea. On the contrary, it might also be asymptomatic. Chronic coronary syndrome has the potential to develop into acute coronary syndrome [4]. When a patient suffers from an acute coronary syndrome, it will often manifest as myocardial infarction or unstable angina with recent changes of the symptoms, such as increasing or persistent chest pain. Eventually, it can lead to heart failure or cardiac arrest [5]. At the moment, there is no standardized quantifiable method to discriminate different stages of CAD. Historically, the severity of CAD was assessed by invasive angiography, and the disease was categorized into single-vessel coronary artery disease (SVCAD), double-vessel coronary artery disease (DVCAD), and triple-vessel coronary artery (TVCAD) based on the number of large vessels that were affected [6]. More recent approaches employed non-invasive imaging techniques that allowed for a more detailed description of the disease progress using a combination of different parameters.

Min et al. [7] used computed tomography angiography (CCTA) and quantitative computed tomography (QCT) to determine total plaque volume (TPV, mm^3^) and percent atheroma volume (PAV, %). Based on these data, they found that the atherosclerotic plaque burden by QCT was related to stenosis severity and extent as well as ischemia and they proposed a four-tiered staging: Stage 0 (Normal, 0% PAV, 0 mm^3^ TPV), Stage 1 (Mild, >0–5% PAV or >0–250 mm^3^ TPV), Stage 2 (Moderate, >5–15% PAV or >250–750 mm^3^ TPV), and Stage 3 (Severe, >15% PAV or >750 mm^3^ TPV).

The Coronary Artery Disease-Reporting and Data System (CAD-RADS™ 2.0) is aimed at establishing a standardized reporting system for patients undergoing CCTA with the additional goal to also guide the resulting treatment regimen [8]. The CAD-RADS classification is based on the assessment of ischemia by CT fractional-flow-reserve or myocardial CT perfusion, plaque burden, and additional modifiers including high-risk plaque, stent, or graft and comprised five stages that allow for a more granular assessment of CAD severity. Finally, several studies have been conducted to apply machine learning techniques on clinical and biometric data to assess CAD. So far, accuracy rates of about 80% for systems without human expert input and about 85% for systems with human expert input have been achieved, demonstrating that an AI-driven CAD classification and identification is feasible; however, human input is still needed. So far, no standardized method has been established. An overview of these works is given in [9].

Important risk factors associated with the development of CAD are smoking, diabetes, hyperlipidemia, high systolic blood pressure, high body mass index (BMI), sedentary behavior, heavy drinking, age, and family history [10,11,12,13,14,15].

### 1.2. Mechanisms of CAD Development

CAD is a complex condition, and several parameters influence its development [16]. Atherosclerosis is an inflammatory process with free radicals, cytokines, lipids, growth factors, prothrombotic, endocrine, and paracrine factors [17]. In the early stages of atherosclerosis, endothelial dysfunction will often be present. Besides contributing to the atherosclerotic process, it may also lower the coronary flow reserve, as the endothelium at some point loses its ability to dilate when needed [18]. Endothelial cells play an important role in protecting against the formation of plaques. When the vessel wall disrupts, oxidized low-density lipoprotein (LDL) particles enter, followed by inflammatory cells such as monocytes, that transform into macrophages. The macrophages absorb oxidized LDL particles, which transform them into foam cells. Ultimately, the plaque forming in the tunica intima consists of inflammatory cells, such as the transformed macrophages, lipid molecules, smooth muscle cells, and extracellular matrix. In the beginning, the plaque will be stable, but as it develops it will become unstable and prone to rupture [19]. Hemodynamic forces, such as shear stress, are attributed to plaque formation and progression to unstable plaques. Stone et al. found that low local endothelial shear stress is associated with an increased plaque burden [20], and Xie et al. reported that low shear stress induced endothelial cell apoptosis and monocyte adhesion [21]. It has also been discussed whether stenosis or the entire plaque burden were the most important risk parameter for cardiovascular disease (CVD) events. Mortensen et al. found that it is the plaque burden, and not the stenosis, that is the main risk factor [22].

### 1.3. Current Treatment of CAD

When treating coronary artery disease, reducing the risk of death and ischemic events is the main goal [23]. Patient education and lifestyle therapy by risk factor management are used as secondary prevention for cardiac events. Lifestyle interventions include the avoidance of smoking and substance abuse, weight management, treatment of hyperlipidemia, diabetes, and hypertension, as well as reducing alcohol consumption, eating healthily, and increasing physical activity. Besides lifestyle therapy, medical therapy is an important part of the treatment. Among other prescription drugs, the treatment often involves aspirin, beta-adrenergic receptor blockers, and statins. The third option for treatment is invasive and consists of percutaneous coronary intervention or coronary artery bypass grafting [4].

### 1.4. Vitamin D

Vitamin D deficiency is a prevalent condition around the world. It is known to be the cause of bone disorders, but it may also be a risk factor for other diseases such as diabetes and cardiovascular disease [24].

**Figure 1:**
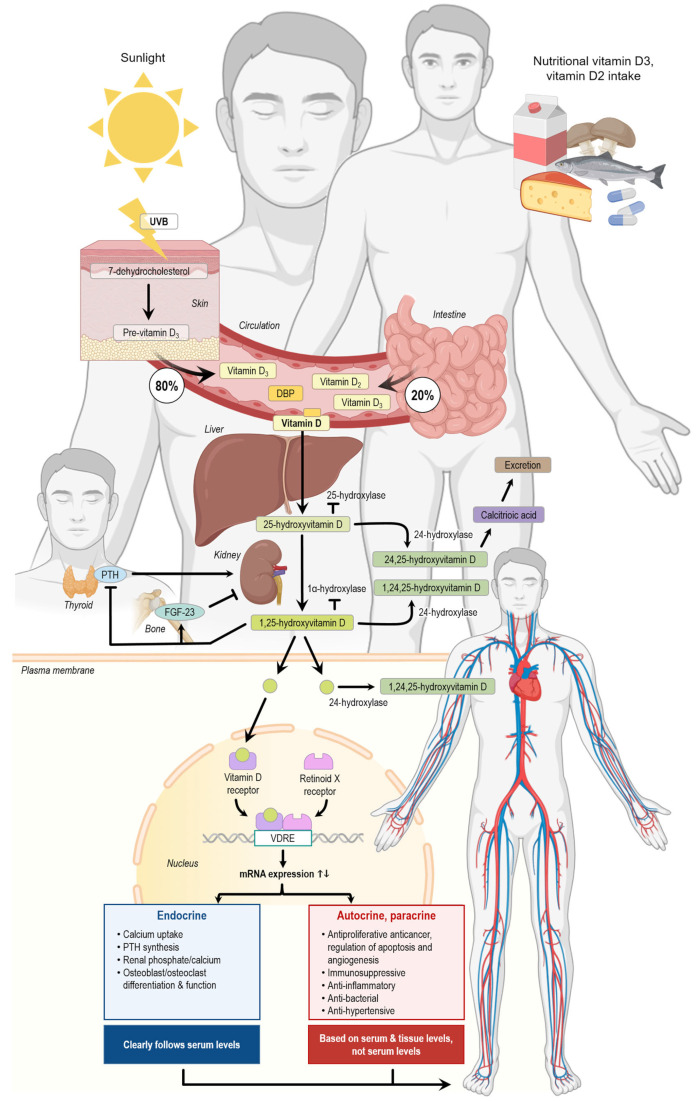
Vitamin D production, metabolization, and action in the human body. DBP, vitamin D-binding protein; FGF-23, fibroblast growth factor 23; PTH, parathyroid hormone; UVB, ultraviolet B radiation, VDRE, vitamin D response element. The arrows indicate the single steps of the vitamin D metabolism. The figure was created using elements from Biorender.

Vitamin D is a fat-soluble vitamin that can be synthesized in the body itself but can also be added to the body through dietary intake (Figure 1). The term vitamin D includes both vitamin D2 (ergocalciferol) and vitamin D3 (cholecalciferol). Cholecalciferol is synthesized in the skin after exposure to UV-B radiation from sunlight or absorbed through the small intestine after dietary intake. Ergocalciferol, on the other hand, is only taken up through the diet. The cholecalciferol from the skin is transported to the liver through the blood, bound to D-vitamin binding protein (DBP). In the liver, cholecalciferol is hydroxylated by CYP-enzymes to 25(OH)D. The same process is happening to ergocalciferol and cholecalciferol from dietary intake, which are transported in the blood mainly in chylomicrons. Afterwards, 25(OH)D is transported to the kidney either freely or bound to D-vitamin binding protein or albumin. In the kidney, 25(OH)D is hydroxylated by 1α-hydroxylase to 1,25(OH)_2_D. 1,25(OH)_2_D which is known to regulate gene transcription by binding to its nuclear receptor (VDR). Furthermore, it can activate several non-genomic pathways [25]. The vitamin D receptor can be found at many different locations throughout the body, including the cardiovascular and immune system, which advocates for the broad effect of the hormone [26]. Indeed, a correlation between low vitamin D serum levels and cardiovascular disease has been reported [27]. There is no consensus on the threshold of vitamin D deficiency, but there is consensus that serum 25(OH)D below 25 nmol/L should be avoided [24].

### 1.5. The Effect of Vitamin D Supplementation on the Ca^2+^ Metabolism

Calcium metabolism plays an important part in maintaining a healthy body as it has been linked to peak bone mass. The first important role of calcium metabolism is absorption of calcium from the diet through the intestines. This occurs mostly through a saturable transport mechanism across the small intestine, but there is also constant non-saturable transportation [28]. Vitamin D regulates calcium absorption through the enterocytes by upregulating the transcription of proteins such as TRPV6 and PMCA1B, which are involved in the transcellular transport of calcium [29]. Vitamin D is also important for renal resorption of calcium in the distal tubule [30]. Besides regulating the absorption and resorption of calcium, vitamin D also acts on osteoblasts and osteocytes to activate osteoclasts through RANKL. Activation of osteoclasts leads to resorption of bone and thereby to an increase in serum calcium [31]. Moreover, parathyroid hormone (PTH) influences calcium metabolism by increasing the synthesis of active vitamin D. PTH also increases osteoclast formation [32].

### 1.6. Effect of Vitamin D on Cardiac Physiology

Fibroblast growth factor 23 (FGF-23) is one of the hormones regulating calcium and phosphorus metabolism. It is therefore closely regulated by PTH and vitamin D levels. Faul et al. reported that an increased level of FGF-23 in patients with chronic kidney disease had the potential to independently increase the risk of developing left ventricular hypertrophy. Furthermore, they found fibroblast growth factor receptor (FGFR) activation to be an important part of the pathology, as binding of the receptor activates the PLCγ (Phospholipase C gamma)-calcineurin-NFAT (nuclear factor of activated T-cells) pathway [33].

Another function of vitamin D is to modulate the transient receptor potential channel (TRPC), which is a mechanosensitive receptor found in cardiomyocytes and the cardiovascular system. Stratford et al. showed that persistent vitamin D deficiency in mice lowers their ability to modulate the heart’s response to cardiac stress causing mechanical changes of the heart. Furthermore, they demonstrated that the altered stress response, leading to cardiac changes, was caused by TRPC6, which is normally suppressed by vitamin D [34]. Pilhouze et al. investigated the cardioprotective effects and the use of vitamin D3 as secondary prevention on cardiac remodeling in diabetic rodents. They found that vitamin D3 lowered left ventricular hypertrophy and alleviated cardiac remodeling and fibrosis caused by type 2 diabetes mellitus. Furthermore, they reported a protective activity of vitamin D3 against lipotoxicity and proposed a reduction in ceramides and diacylglycerol (DAG) levels in the myocardium as one of the possible mechanisms of the cardioprotective effect of vitamin D [35]. 

Another study conducted by Ivkovic et al. investigated whether vitamin D directly affects the cardiac energy metabolism and contraction–relaxation process. It was found that vitamin D decreased the expression of malonyl-CoA-decarboxylase, while increasing anti-phospho-acetyl-CoA, both of which regulate the expression of malonyl-CoA, an important factor for beta-oxidation. They also detected that the ryanodine receptor was decreased in the vitamin D-treated group and that the expression of the (sarcoplasmic/endoplasmic reticulum Ca2+-ATPase isoform 2) (*SERCA2*) was increased along with changes in other important proteins for energy metabolism and regulation of the contraction–relaxation mechanism of the myocardium [36]. Figure 1 shows an overview of the effects of vitamin D on the human body.

## 2. Results

### 2.1. Vitamin D and Hypertension

Hypertension is a significant risk factor for the development of cardiovascular disease. Hypovitaminosis D has been associated with the development of hypertension. A recent study tested the effect of a knockout of vitamin D receptor in myeloid cells in mice. The animals developed an increased mean arterial blood pressure. Vitamin D deficiency increased the vascular and renal infiltration of immune cells, such as macrophages. Juxtaglomerular infiltration of macrophages elevated the renin–angiotensin system by stimulating the juxtaglomerular cells, by secreting *miR-106b-5p*. Secretion of *miR-106-5b* is endoplasmatic reticulum (ER)-stress-induced, and it was shown that it was increased by the vitamin D receptor deletion. In addition, renal infiltration of macrophages reduced nitric oxide (NO) by increasing reactive oxygen species (ROS). Thereby, a reduction in renal perfusion occurred, which increased the renin secretion. The authors also demonstrated that restoring the receptor by transplanting the mice with wild-type bone marrow prevented hypertension [37].

### 2.2. Vitamin D and Atherosclerosis

The development of foam cells is an important part of the atherosclerotic process. Kumar et al. found that vitamin D could help preventing the formation of foam cells by enhancing the macrophages’ ability for lipid autophagy, which is otherwise prevented by the oxidized LDL molecules. They tested the impact of vitamin D on autophagy in both macrophages from mice and humans. The rescue of impairment of autophagy and lipid degradation, and thus salvage of macrophages from turning into foam cells, was due to the VitD3-VDR-tyrosine-protein phosphatase non-receptor type 6-(PTPN6)-pathway. PTPN6 is a tyrosine phosphatase that plays a role in vascular homeostasis. The authors demonstrated that vitamin D3 treatment leads to an increase in PTPN6 expression. The signal transducer and activator of transcription 3 (STAT3) is known to inhibit autophagy. It was reported that the activation of STAT3 was inhibited during vitamin D treatment via dephosphorylation caused by PTPN6. This mediated the expression of the autophagy proteins beclin-1 (BECN1) and autophagy protein 5 (ATG5). Furthermore, it was reported that vitamin D and PTPN6 regulated the mitogen-activated protein kinase 1 (MAPK1) activation, which is also important for autophagy. Vitamin D3 treatment therefore led to a decrease in foam cell formation [38].

Vitamin D also alters the lipid profile of the blood. Elseweidy et al. tested the effect of vitamin D3 on diabetic hyperlipidemic rats. They found that it is cardioprotective, as the treatment reduced LDL particles and triglycerides while increasing high-density lipoprotein (HDL) particles, achieving a more favorable lipid profile. The authors hypothesized that the effect was caused by an altered hepatic lipid metabolism. They also investigated the aortic tissue. The control group showed a subintimal infiltration of foamy cells, thickened intima, and plaques. The vitamin D3-treated rats revealed fewer foam cells and an irregular intima. They also observed an anti-inflammatory action of vitamin D3, as it reduced CRP levels [39].

### 2.3. Vitamin D and Vascular Reactivity

A study on male rats investigated the effects of vitamin D on the reactivity of the coronary arteries and the receptor expression in the intramural segments. The animals were divided into two groups: One control group with optimal vitamin D levels and one vitamin D-deficient group. The authors observed a significant difference in the inner radius of the arteries as the radius of the vitamin D-deficient group was smaller (*p* < 0.01). When measuring the constriction ability of the coronary arteries, they tested the response of thromboxane A2, as under normal circumstances it functions as a vasoconstrictor. It was observed that thromboxane-induced contraction of the arteries was significantly decreased (*p* < 0.05), and expression of the receptor was also reduced in the vitamin D-deficient group. When testing for 17-β-estradiol, testosterone, adenosine, and insulin-induced relaxation, the arterioles in the vitamin D-deficient rats showed less vasodilation in response to 17-β-estradiol (*p* < 0.05) and testosterone (*p* < 0.05). The vasodilation mediated by adenosine and insulin remained unchanged. A diminished expression of the estrogen receptor α was on the border of being significant (*p* = 0.0571). There was no difference in the androgen receptor expression. A reduction of the thromboxane receptor might be the reason for the diminished effect of thromboxane A2 on vasoconstriction, whereas the decreased effect of the sex hormones appears to be functional. The impaired reactivity of the vessel to thromboxane and different sex hormones could cause an inadequate coronary perfusion and thereby increased cardiovascular risk [40].

### 2.4. Vitamin D and Myocardial Infarction

Vitamin D exhibits a protective potential following myocardial infarction. Wei et al. conducted an animal study using mice with induced acute myocardial infarction. They found that treatment with vitamin D3 decreases cardiac dysfunction caused by acute myocardial infarction (AMI). The effect of vitamin D3 was due to the inhibition of the phosphatidylinositol 3-kinase/ protein kinase B (Ak strain transforming)/mammalian target of rapamycin (PI3K/AKT/mTOR; PAM) pathway, which enhances autophagy and inhibits apoptosis of cardiomyocytes [41].

Mehdipoor et al. also investigated the effect of vitamin D3 in myocardial infarction. They used rats to test the effect of vitamin D3 supplements and aerobic resistance training and found vitamin D to prevent the progression of fibrosis and improve the ejection fraction. TGF-β1, Smad2/3, and collagen are all factors promoting cardiac fibrosis. The authors demonstrated an increase in the mediators in case of a myocardial infarction. The treatment with vitamin D3 reduced the expression of transforming growth factor beta 1 (TGF-β1), smad2/3, and collagen and thus the progression of fibrosis. The effect was strongest when the vitamin D supplementation was combined with aerobic-resistant training [42]. Another study investigated the cardioprotective effects of calcitriol in mice with myocardial infarction and found cardioprotective effects in a dose-dependent manner. It was also demonstrated that calcitriol could restore vascular dysfunction after myocardial infarction as well as reverse pathological aortic remodeling. It was hypothesized that the cardioprotective role of vitamin D is due to the modulation of the nuclear factor ‘kappa-light-chain-enhancer’ of activated B-cells (NF-κB)-pathway, which normally has its role in producing inflammatory cytokines. The authors found that the NF-κB-pathway was indeed diminished by calcitriol, while the (interleukin 10) *IL10* gene expression was increased, which suppressed cardiac inflammation [43].

### 2.5. Randomized Controlled Trials

Table 1 gives an overview of the designs and the results of clinical trials studying the influence of vitamin D on cardiovascular disease since 2020.

## 3. Discussion

The DO-health trial studied the impact of vitamin D3, omega-3, and a strength exercise program on cardiovascular disease in older adults, as age is an important risk factor of developing cardiovascular disease. The science team did not find any effect of the vitamin D treatment regarding the patient’s lipid profile or regarding the risk of major cardiovascular events (MACEs). An important consideration of the study was that participants were largely vitamin D-replete and, even though they were older adults (≥70 years), generally healthy. This might have made the intervention less effective as it might not have been needed [54].

Hasific et al. investigated the effect of vitamin K2 and vitamin D on coronary artery calcification and plaque development. The participants had to have an aortic valve calcification (AVC) score ≥ 300, leaving them at risk of cardiac events. A stratified analysis showed that participants with a coronary artery calcification (CAC) score ≥ 400 AU had a significantly lower progression of coronary artery calcification compared to the placebo group (*p* = 0.047), suggesting that the intervention was beneficial for the patients at higher risk of CVD. However, the intervention arm involved both vitamin K2 and vitamin D3 and it was therefore not possible to conclude whether vitamin D3 alone had an effect [50]. Sadeghi et al. investigated participants with ischemic heart disease and hypovitaminosis D regarding vitamin D supplements effect on CRP and lipids. They found HDL to be increased and triglycerides (TGs) to be decreased in the intervention group, but the changes did not differ significantly from the changes in the placebo group, even though serum levels of vitamin D were increased significantly in the intervention group compared to the placebo group. The CRP levels did not change significantly. The changes in the placebo group may be due to cardiac medication during the period. Furthermore, the study had a small population and a short follow-up time, which also could have influenced the results [56].

Johny et al. studied whether vitamin D supplementation in participants with type 2 diabetes and vitamin D deficiency would influence platelet activation, the glycemic profile, and systemic inflammation. The intervention did not influence the glycemic profile, but it showed that vitamin D supplementation reduced PAC-1, P-selectin, platelet factor 4, and 11-dehydrotrhromboxane B2 expression, which are all markers for platelet activation. In addition, the platelet aggregate formation with monocytes was reduced. A concomitant ex vivo study showed lower intracellular ROS levels. A decrease in the expression of cytokines was also found. Platelet activation, inflammation, and oxidative stress are important contributors to CAD development and the treatment and therefore showed a protective effect [57].

Moreover, other studies showed that vitamin D3 is anti-inflammatory and furthermore lipid-lowering in diabetic mice, in combination with results from the clinical trials demonstrating a possible effect of vitamin D3 supplements on type 2 diabetic patients [39].

Imanparast et al. also studied a type 2 diabetic population. They investigated whether vitamin D3 and chromium picolinate could reduce endothelial dysfunction. The clinical trial was carried out in a population with vitamin D deficiency. Vitamin D reduced homocysteine and malondialdehyde. Both homocysteine and malondialdehyde are associated with promoting endothelial dysfunction by oxidative stress. The study thereby suggested that vitamin D can be protective for the atherosclerotic process and CAD. The trial had a small sample size and dividing it into four categories made it even smaller, which is a limitation of the study [58].

Both Morrone et al. and Limonte et al. examined the impact of vitamin D supplements in patients with impairment of the kidneys, as they are at increased risk of developing cardiovascular disease. Neither of the studies found vitamin D to reduce cardiovascular events [47,53].

The Polycap study investigated whether vitamin D supplements of 60,000 IU of vitamin D3 given monthly influenced skeletal and non-skeletal clinical outcomes in a non-Western population with increased risk of cardiovascular disease. They found no evidence that the supplements altered cardiovascular outcomes, but rather a higher death rate in the vitamin D-supplemented group. This might be a coincidence, as the study was not designed for detecting this endpoint or it might be correlated to the ethnicity of the participants [48]. The D-health study investigated whether a monthly dose of 60,000 IU vitamin D3 in older adults would be beneficial for preventing cardiovascular events. They found the rate of cardiovascular events to be lower in the vitamin D-supplemented group compared to the placebo group, and even more so in a subgroup taking cardiovascular drugs at baseline. Importantly, no significant differences were found [44].

The D-health study may be compared to the DO-health study as the populations were somewhat comparable. A difference between the studies was the intervention method as the DO-health study gave vitamin D3 in a smaller dose daily, whereas the D-health study gave one bigger dose monthly. This could explain why the D-health study demonstrated an effect, even though it was not significant, and the DO-health study did not. On the other hand, the Polycap study also gave one bigger dose monthly but did not discover any treatment effect. Differences in ethnicity in the population groups of course make it difficult to directly compare the studies [48]. As mentioned, the D-health study observed a greater effect among patients taking cardiovascular drugs at baseline. This tendency resembles the findings of Hasific et al. [50], where the patients with greater calcification at baseline had greater effects from the treatment.

Sarhan et al. found that vitamin D supplements can increase the ejection fraction, decrease cardiac fibrosis markers, and decrease the number of participants experiencing major adverse cardiac events (MACEs). They also found certain genetic mutations of the VDR to be associated with the development of heart failure and MACEs. All participants suffered from acute coronary syndrome at baseline [49]. The study advocates that vitamin D might be effectful as an add-on treatment in more advanced disease. Furthermore, knowledge of VDR mutations and their relation to specific cardiac events might be an important subject in future research. Lastly, two randomized control trials (RCTs) cover vitamin D supplements as an add-on treatment before coronary artery bypass surgery to prevent adverse events following the intervention.

Tasdighi et al. investigated whether inflammation-induced heart injury following coronary bypass surgery could be avoided. They included patients that were vitamin D-deficient and treated them 3 days prior to surgery. They found a decreased expression of caspases 2 and 3 in the intervention group compared to the placebo group. Caspases 2 and 3 are biomarkers of myocardial apoptosis. In addition, IL-10 remained elevated in the intervention group. The decrease in apoptosis and inflammation protected the cardiomyocytes [51].

Alirezaei et al. showed that high-dose vitamin D supplementation prior to coronary artery bypass grafting surgery led to a significant reduction in postoperative atrial fibrillation [52].

When assessing the usefulness of long-term vitamin D supplementation to improve cardiovascular outcomes, it is also necessary to consider possible side effects and even toxicity of high-dose regimens. So far, a dose of 4000 IU/day has been considered safe; however, this limit has been questioned recently. Rizzoli [59] performed an in-depth analysis of vitamin D supplementation in different clinical studies and found that dose safety not only depends on the amount of vitamin D alone, but also on the vitamin D status, the regimen, the outcome, and possibly the age and sex of the recipient. The author proposes a dose of 800–1000 IU/daily of vitamin D or 10 μg/day of calcifediol, respectively, as safe. Considering this threshold, most studies discussed in this review exceed the upper limit of safe vitamin D dosage. In the light of the very limited effects of vitamin D supplementation on CAD, this raises the question of whether long-term side effects might outweigh the benefits of this approach. So far, most high-vitamin D trials were not designed to address this point; therefore, future trials should be suitably adapted.

While there are extensive in vitro and animal studies exploring the effects of vitamin D on cardiovascular tissues and CAD-related pathways, only few biochemical analyses have been conducted in the clinical trials reviewed here. Replicating and/or validating the results from the preclinical experiments in a randomized controlled study would help estimate how far they can be translated into the clinical setting and whether further studies are justified.

It is striking that, in all the presented trials in this review, as well as in other meta-analyses [60,61], vitamin D had no or only minuscule beneficial effects on cardiovascular endpoints irrespective of target groups, baseline vitamin D status, and vitamin dosage or regimens. Of note, most studies did not rule out prior and parallel vitamin D supplementation of <400–800 IU/d, so essentially all participants could be considered vitamin D-replete at the start of the trials. It can be speculated that vitamin D does not exhibit a linear dose–response regarding clinical outcomes, but rather that there exists a “threshold” around the physiological level of vitamin D, above which no or only minute additional effects and benefits can be observed. A study with vitamin D-deficient participants could help clarify this point; however, ethical considerations (washing-out prior study start) might be problematic.

One exception is the study by Sarhan et al. [49]. Besides administering relatively high doses, which poses a certain risk, as discussed earlier, they varied the treatment regimen according to the blood vitamin D level of the participants and achieved a statistically significant reduction in MACEs. Their findings also seem to suggest that the VDR gene polymorphisms are responsible for susceptibility to cardiovascular complications in CAD and that genetic and epigenetic typification of patients might improve the success rate of vitamin D therapy in small defined subpopulations. It is possible that these effects were diluted and masked by baseline noise in large population studies.

Lastly, two trials studied vitamin D in combination with omega-3 fatty acids (DO-HEALTH and VITAL). None of these studies found a substantial synergistic effect between the two drugs on CAD. This is in accordance with earlier analyses, which also failed to detect a benefit from vitamin D/omega-3 [62,63,64]. In the AVADEC trial, Hasific et al. observed no significant differences in coronary artery calcification (CAC) in participants supplemented with vitamin K2 and D versus placebo overall; however, they found a significantly reduced progression in patients with CAC scores >400. This finding is a further indicator that vitamin D supplementation (in combination or not) might not be effective for a large portion of the population but should rather be applied in very specific scenarios.

## 4. Materials and Methods

**Figure 2:**
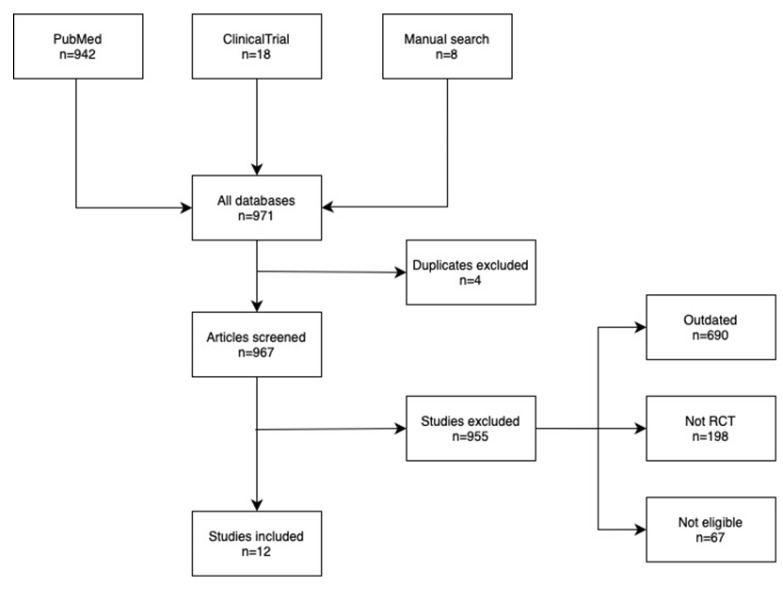
Flowchart of the selection process of the discussed studies.

The research for this review was conducted by using the databases PubMed (https://pubmed.ncbi.nlm.nih.gov) and ClinicalTrials.gov (https://www.clinicaltrials.gov), accessed on 12 May 2025. The used search terms were: cholecalciferol, “vitamin D”, “Myocardial Infarction”, and “coronary artery disease”, as well as their combinations ((((“Cholecalciferol”[Mesh]) OR (“vitamin D”[Mesh])) OR (Cholecalciferol)) OR (“vitamin D”)) AND ((((“Myocardial Infarction”[Mesh]) OR (“Coronary Artery Disease”[Mesh])) OR (“Myocardial infarction”)) OR (“Coronary artery disease”)). The search yielded 942 articles in PubMed. In ClinincalTrials.gov the search for the condition was “Coronary Artery Disease” OR “Myocardial Infarction” and for treatment “vitamin D” OR Cholecalciferol. This yielded 18 results. Randomized controlled trials with vitamin D supplements as the intervention arm and testing on populations with coronary artery disease or in high risk of developing it and articles published in the year 2020 or later were included. Studies that were not written in English, studies on populations younger than 18 years, and studies that had not posted any results were excluded. Figure 2 illustrates the process of finding the articles.

## 5. Conclusions

The clinical trials conducted between 2020 and 2024 provide ambiguous results concerning the potential of vitamin D as an add-on for the treatment for coronary artery disease, even though research papers are providing evidence of protective effects of vitamin D. This could be due to the difference between animal and human anatomy and physiology but might also be due to a lack of clinical trials focusing on specific subgroups.

The clinical trials and research papers showed vitamin D supplement’s possible effect in type 2 diabetic patients as a lipid-lowering and anti-inflammatory drug, as well as for reducing endothelial dysfunction. The research also showed the potential of vitamin D as an add-on treatment in patients with more developed coronary artery disease.

On the contrary, vitamin D supplements did not seem to have an effect in the broader and generally healthy older population, even though their age put them at risk of CVD. Furthermore, high doses of vitamin D before coronary bypass surgery might also be preventive of cardiac events following the intervention.

Even though it seems possible that some subpopulations may benefit from vitamin D supplements, the clinical trials differ greatly from each other and are not easy to compare, which makes it difficult to determine the efficacy of vitamin D as an add-on treatment. Further research should be conducted on this matter.

## Figures and Tables

**Table 1:** An overview of the relevant clinical trials conducted since 2020.

Study, Identifier	Design	Intervention	Results
Vitamin D supplementation and major cardiovascular events: D-health randomized controlled trial [44,45,46] ACTRN12613000743763	Phase 3, randomized, double blinded, placebo-controlled trial *n* = 21,315 (60–84 years old) follow-up: 5 years endpoint: major cardiovascular event (Myocardial infarction, stroke or coronary revascularization)	One monthly dose of 60,000 IU vitamin D vs. soya oil placebo data	The rate of major cardiovascular events was lower in vitamin D group than in placebo group (HR = 0.91, 95% CI: 0.81–1.01) The rate of major cardiovascular events was even lower in patients taking cardiovascular drugs (HR = 0.84, 95% CI: 0.74–0.97, *p* = 0.12) The hazard ratio was lower when looking at incidents of myocardial infarction (HR = 0.81, 95% CI = 0.67–0.98) and coronary revascularization (HR = 0.89, 95% CI: 0.78–1.01) However, all differences were not statistically significant
Calcifediol supplementation in adults on hemodialysis: a randomized controlled trial [47] NCT01457001	Phase 3, multicenter, randomized controlled trial *n* = 284 (≥18 years) follow-up period = 24 months Endpoint: composite of nonfatal myocardial infarction, nonfatal stroke and death	40 µg calcifediol 3 times a week vs. no additional treatment	Calcifediol did not have effect on cardiovascular death (HR: 1.06, 95% CI: 0.41–2.74) or nonfatal myocardial infarction (HR: 0.20, 95% CI: 0.02–1.67) The intervention did not show an effect on the composite of nonfatal myocardial infarction, nonfatal stroke, and death (HR = 1.03 95% CI: 0.63–1.67)
The International Polycap Study 3 (TIPS-3) [48] NCT01646437	Phase 3, double-blind, placebo-controlled, randomized trial *n* = 5670 (men aged ≥ 50 years and women aged ≥ 55 years) Mean follow-up: 4.6 years Endpoint: fracture and composite of CV death, myocardial infarction stroke, cancer, fracture or fall	One monthly dose of 60,000 IU of vitamin D3 vs. placebo	High dose vitamin D did not reduce non-skeletal outcomes (HR = 1.13, 95% CI: 0.93–1.37, *p* = 0.22) More people in the vitamin D group died (HR = 1.29, 95% CI: 1.03–1.61, *p* = 0.03)
Impact of Vitamin D supplementation on the Clinical Outcomes and Epigenetic Markers in Patients with Acute Coronary Syndrome [49] Trial registration not given	Randomized controlled trial *n* = 250 Follow up: 2 years Endpoints: cardiac fibrosis markers, echocardiographic parameters, epigenetic markers	Intervention arm: Vitamin D based on serum vitamin D level at baseline. The intervention was: 50,000 IU/week for 8 weeks followed by 10,000 IU/week for 4 months if serum vitamin D was 12 ng/mL and 10,000 IU/week for 6 months if serum vitamin D was above 12 ng/mL	The intervention lowered ejection fraction (*p* = 1.1 × 10^−4^), end systolic volume (*p* = 0.0075), and end diastolic volume (*p* = 0.002) There was a decrease in cardiac fibrosis markers There were fewer events of MACE (*p* = 0.043) Taq I (rs731236) was a predictor of heart failure, Bsm I (rs1544410) was a predictor of MACE, Fok I (rs2228570) protected against MACE
The Aortic Valve DECalcification Trial (AVADEC) [50] NCT03243890	Randomized, controlled, double blinded trial *n* = 304 (men, 65–75 years old) Follow-up time: 2 years Endpoint: absolute change in CAC score and changes in plaque volume	Menaquinone-7 720 µg/day including the recommended daily dose of vitamin D (25 µg/day) vs. placebo	There was no overall reduction in mean CAC progression (mean difference = 51 AU, *p* = 0.089) Participants with CAC scores ≥400 AU had a smaller progression in CAC (380 AU vs. 288 AU, *p* = 0.047) Participants with statin use also had a smaller progression in CAC (*p* = 0.048)
Can Vitamin D Reduce Heart Muscle Damage After Bypass Surgery? [51] NCT04323852	Phase 4, double blind, randomized, placebo-controlled trial *n* = 70 (≥18 years) follow-up: 30 days Endpoint: caspases 2, 3 and 7 activity, IL-10 serum level, IGF-I serum levels and pro-BNP	3 doses of vitamin D (50,000 U) a day for 3 days before surgery vs. placebo	Lower average number of caspases 2 and 3 in the vitamin D group (*p* = 0.006) Increased levels of IL-10 in the vitamin D group before surgery (*p* = 0.001). It remained elevated after surgery compared to the intervention group (*p* < 0.001) No difference in pro-BNP
Effect of preoperative Vitamin D on postoperative atrial fibrillation incidence after coronary artery bypass graft [52] IRCT20230506058103N1	Phase 3, randomized controlled clinical trial *n* = 246 follow-up = 5 days Endpoints: POAF up to 5 days after surgery, duration of hospitalization, duration of intubation	Starting 3 days before the surgery, patients receive 50,000 units of vitamin D three times a day vs. placebo	There was no significant difference in the duration of the hospital stay (*p* = 0.975) or intubation period (*p* = 0.886) Decrease in POAF incidence in the intervention group (*p* = 0.003)
Vitamin D and Omega-3 Trial (VITAL) [53] NCT01169259	Phase 3, double-blind, placebo-controlled trial *n* = 15,917 (men ≥ 50 years and women ≥ 55 years) Endpoint: major cardiovascular events, invasive cancer	Daily doses of: 2000 IU vitamin D3 + 840 mg of marine omega-3 fatty acids vs. 2000 IU vitamin D3 + fish oil placebo vs. vitamin D placebo + 840 mg of marine omega-3 fatty acids vs. vitamin D placebo + fish oil placebo	There was no significant difference in major cardiovascular events between the intervention and control group There was no significant interaction between eGFR, vitamin D, and major cardiovascular events
DO-HEALTH/Vitamin D3—Omega3—Home Exercise—Healthy Ageing and Longevity Trial (DO-HEALTH)) [54,55] NCT01745263	Phase 3, double-blind, randomized placebo-controlled trial *n* (biomarkers) = 2157 n (MACE) = 2089 age ≥ 70 years Endpoint: lipid and CVD biomarkers, incident hypertension, and major cardiovascular events. Follow-up: 3 years	2000 IU/d of vitamin D3, 1 g/d of omega-3s, and a strength-training exercise program vs. vitamin D3 and omega-3s vs. vitamin D3 and vs. vitamin D3 alone vs. omega-3s and exercise vs. omega-3s alone vs. exercise alone vs. placebo	Vitamin D3 did not change the lipid profile significantly Vitamin D3 supplements showed no effect on MACE (HR = 1.37, 95% CI: 0.88–2.14), there were also no effects on hypertension (HR = 1.05, 95% CI: 0.76–1.44)
The effect of vitamin D deficiency treatment on lipid profile in ischemic heart diseases: a double blinded randomized clinical trial [56] IRCT20200905048622N1	Phase 3, double-blind, randomized controlled trial *n* = 44 (40–65 years) Follow-up time: 5 weeks Endpoints: serum levels of TGs, LDL-C, HDL-C, TC and CRP	50,000 IU vitamin D3 weekly up to 2 months vs. placebo	A significant increase in HDL (*p* = 0.048) and a significant decrease in TG (*p* = 0.008) in the intervention group No correlation between vitamin D and CRP levels
Vitamin D supplementation modulates platelet-mediated inflammation in subjects with type 2 diabetes: A randomized double-blind, placebo-controlled trial [57] CTRI/2019/01/016921	Randomized, double-blind, placebo-controlled trial *n* = 59 (25–65 years) follow-up = 12 months endpoint: platelet activation, platelet-immune-cell aggregation, immune profile, vitamin D metabolite levels	Vitamin D 60,000 IU/week for 3 months followed by 60,000/month for 9 months	No improvement of glycemic control or a difference in immune cells between the groups Platelet activation was reduced, and platelet-immune cell aggregates were altered (*p* < 0.05) Reduced levels of cytokines such as IL-18, TNF-alpha, IFN-gamma, etc. Reduction in intracellular reactive oxygen species
Simultaneous use of chromium picolinate and vitamin D reconstruction or prevention of endothelial dysfunction in patients with type 2 diabetes by examining the pattern of changes in metabolic markers of oxidative stress, inflammation, and endothelial dysfunction [58] IRCT2017052034038N1	Phase 3, randomized, single-blind, placebo-controlled trial *n* = 92 (25–60 years) Follow-up time: 4 months Endpoints: Hct, MDA, total antioxidant capacity, total thiol groups, vascular cell adhesion molecule-1, plasminogen activator inhibitor-1	6 months of: 400 mg of chromium picolinate daily vs. 50,000 IU vitamin D per week vs. combination of 50,000 IU per week vitamin D and 400 mg of chromium picolinate per day vs. placebo	Vitamin D3 significantly reduced homocysteine Vitamin D3 significantly changed the mean concentration of MDA Vitamin D3 significantly decreased the expression of PAI-1

HR = Hazard ratio, CI = confidence interval, CV death: cardiovascular death, CAC = coronary artery calcification, IL-10 = interleukin 10, POAF: postoperative atrial fibrillation, eGFR = estimated glomerular filtration rate, MACE: major cardiovascular events, CRP = C-reactive protein, TG = triglycerides, LDL-C = low-density lipoprotein cholesterol, HDL-C = high-density lipoprotein cholesterol, TC = total cholesterol, IL-18 = interleukin 18, TNF-alpha = tumor necrosis factor, IFN-gamma = interferon gamma, Hct = homocysteine, MDA = malondialdehyde, PAI-1 = plasminogen activator 1.

## Abbreviations

The following abbreviations are used in this manuscript:

AMI	Acute myocardial infarction
AVC	Aortic valve calcification
BMI	Body mass index
CAC	Coronary artery calcification
CAD	Coronary artery disease
CRP	C-reactive protein
CVD	Cardiovascular disease
DAG	Diacylglycerols
ER	Endoplasmic reticulum
FGF-23	Fibroblast growth factor 23
FGFR	fibroblast growth factor receptor
HDL	High-density lipoproteins
LDL	Low-density lipoprotein
MACE	Major cardiovascular events
NO	Nitric oxide
POAF	Postoperative atrial fibrillation
PTH	Parathyroid hormone
RCT	Randomized controlled trial
ROS	Reactive oxygen species
TG	Triglycerides
TRPC	Transient receptor potential channel
VDR	Vitamin D receptor
